# Measurement Invariance in the Assessment of Mood Between American and Mexican Community Studies

**DOI:** 10.1093/geroni/igab046.2476

**Published:** 2021-12-17

**Authors:** Manuel Herrera, Daniel Paulson

**Affiliations:** University of Central Florida, Orlando, Florida, United States

## Abstract

The Health and Retirement Study (HRS), a principal source for American public health research, has numerous global sister studies. Harmonization efforts seeking to establish measurement equivalence amongst these various datasets, is a critical prerequisite to cross-cultural research. Given well-known cultural variability in depressive symptom endorsement, the purpose of this study was to assess measurement invariance in a brief mood measure used in the HRS and the Mexican Health and Aging Study (MHAS). Total sample size using both groups was 15,319 participants (10,931 HRS; 4,388 MHAS) who were 65 and older from Waves 6 to 13 in the HRS and Waves 1 to 4 in the MHAS. MPlus Version 8.4 was used to conduct CFA analyses of measurement invariance. A contemporary approach with categorical data calls for examining threshold invariance first while establishing configural invariance, before examining invariance tests of thresholds, loadings, and intercepts in a second step. Results were that measurement invariance was not supported in this series of two steps with four out of six indices showing model fit in the first model and none of the indices showing model fit in the second model. These findings implied that there were differences in ways of responding to the brief mood measure between HRS and MHAS participants at the conceptual level. Thus, comparisons based on these measures may result in misleading findings and should be interpreted very conservatively. This study adds to the growing body of literature guiding harmonization efforts from the Program on Global Aging, Health and Policy.

